# Biological Response Induced in Primary Human Gingival Fibroblasts upon Exposure to Various Types of Injectable Astringent Retraction Agents

**DOI:** 10.3390/ma14082081

**Published:** 2021-04-20

**Authors:** Danuta Nowakowska, Julita Kulbacka, Joanna Wezgowiec, Anna Szewczyk, Dagmara Baczynska, Marek Zietek, Wlodzimierz Wieckiewicz, Jolanta Saczko

**Affiliations:** 1 Department of Prosthetic Dentistry, Wroclaw Medical University, 50-425 Wroclaw, Poland; danuta.nowakowska@umed.wroc.pl (D.N.); wlodzimierz.wieckiewicz@umed.wroc.pl (W.W.); 2 Department of Molecular and Cellular Biology, Wroclaw Medical University, 50-556 Wroclaw, Poland; julita.kulbacka@umed.wroc.pl (J.K.); a.szewczyk@umed.wroc.pl (A.S.); dagmara.baczynska@umed.wroc.pl (D.B.); jolanta.saczko@umed.wroc.pl (J.S.); 3 Department of Experimental Dentistry, Wroclaw Medical University, 50-425 Wroclaw, Poland; marek.zietek@umed.wroc.pl

**Keywords:** chemical gingival margin retraction/displacement agents, human gingival fibroblasts, biocompatibility, cytotoxicity, cytoskeleton, oxidative stress

## Abstract

Traditional chemo-mechanical retraction/displacement materials can impact the gingival margin tissues. This study was undertaken to analyze biological responses induced in human gingival fibroblasts (HGFs) upon application of injectable astringent-based agents used in the cordless retraction technique. HGFs were exposed to hemostatic agents (five gels, three pastes, and one foam) based on aluminium chloride, aluminium sulphate and ferric sulphate. Changes in cell viability and proliferation were evaluated using an MTT assay and a BrdU assay. The cytoskeleton structure organization (zyxin and F-actin) was visualized by confocal laser scanning microscopy. Oxidative stress was determined using the Griess Reagent System. The RNA expression levels of antioxidant enzymes were quantified by real-time RT-PCR. The statistical significance was evaluated using Student’s t-test and one-way ANOVA with post-hoc Tukey HSD test. The evaluated agents did not downregulate fibroblast viability or proliferation. No significant cytoskeleton reorganization was observed. Only one agent (Expasyl) induced oxidative stress, demonstrated by the increased level of nitrites. Incubation with the studied agents significantly increased the RNA expression of some antioxidant enzymes (SOD1, SOD3, GPX1). However, no significant influence on the expression of SOD2 and HMOX1 was detected. The injectable forms of chemical retraction agents revealed biocompatibility with HGFs, suggesting their potential clinical usefulness in gingival margin retraction.

## 1. Introduction

Various gingival margin retraction/displacement strategies to achieve temporary gingival sulcus dilatation, as well as control moisture and bleeding into the gingival sulcus space, have been proposed in restorative dentistry [1,2,3]. This procedure results in a dry, clean, and visible sulcus space with the aim of the precise imaging of the finish line of prepared teeth, and/or emergence profile of implant structure, via conventional or digital/optical impression [4]. The careful management of the gingival soft tissues also prevents the accidental damage of the teeth surrounding the periodontium, protects the biological space, minimizes the risk of iatrogenic gingival recession, and as a result, prolongs the functional and aesthetic long-term success of indirect and direct dental restoration [5].

At present, the most popular traditional chemo-mechanical methods are based on chemical retraction/displacement agents such as conventional astringents, including hemostatics, as well as vasoconstrictors, including adrenergic drugs, in combination with packing retraction/displacement materials (retraction cords, strips, cotton pellets, tubes, rings, or caps).

The astringents—as inorganic metal salts, including aluminium chloride (AC), aluminium sulphate (AS), ferric sulphate (FS), and rarely, other chemicals or their combination—induce a hemostatic effect via precipitation of the tissues and blood proteins, and coagulation of gingival small vessels. Additionally, they cause temporary constriction of gingival tissues by extracting water from the tissues. Astringents have relativity low cell permeability, and generally, they act as irritants in moderate concentrations and caustics in high concentrations [6,7].

This gingival retraction method, introduced in dentistry in the mid-20th century, uses cords soaked ex tempore in dental practices or preimpregnated by manufacturers with solutions of retraction agents, which are placed into gingival sulcus (cord technique or double-cord technique). The method is claimed to have superior efficiency and popularity [1]. However, numerous adverse, local inflammatory effects on gingival tissues were clinically observed, both reversible and irreversible, including swelling, red marks, discomfort, and pain after retraction procedure and gingiva/tooth hard tissue/or dental restoration discolourations. Additionally, gingival and alveolar crest recession were observed [8,9,10]. This damage has been identified as an after-effect caused by their very low pH-level (<3), caustic properties, and relatively high concentrations. The local damage may also have been caused by the mechanical technique of inserting the gingival retraction media into the gingival sulcus. Therefore, the use of chemical/retraction agents in the more convenient, injectable cordless technique is justified. In the new concept of gingival retraction/displacement, it was proposed to incorporate astringents into the structure of various forms of injectable retraction media such as gels, self-expanding foams, kaolin- or silica-based pastes, polymers matrices, and retracting/impression materials, e.g., silicone elastomers. Such an approach could increase the clinical utility of astringents through improved comfort of use. However, these media injected into the gingival sulcus may expand due to absorption of saliva and crevicular fluid and may release incorporated chemical retraction agents (AC, AS, FS, and eventually others). For this reason, the biocompatibility of these systems should be thoroughly investigated.

Due to discrepancies between the results of different studies evaluating the cytotoxicity of different chemical retraction/displacement agents to periodontal tissues, and sparse information about the biocompatibility of different forms of injectable retraction systems, this research was designed to explain their cellular effects in HGFs. The human gingival fibroblast model is particularly interesting, as it produces most of the extracellular matrix in connective tissues and is an essential part of the wound-healing and reparative regeneration processes. Because of these properties, biological response induced in HGFs upon exposure to various types of injectable astringent retraction agents is an important scientific issue that requires detailed investigation.

## 2. Materials and Methods

### 2.1. Chemical Retraction Agents

Nine commercially available retraction/displacement agents (five gels, three pastes, and one foam) with incorporated astringents were selected for this study. They were divided into 3 categories: seven contained aluminium chloride (AC), one contained aluminium sulphate (AS), and one contained ferric sulphate (FS) in different concentrations. Their characteristics are summarized in Table 1. For this in vitro study, solutions of the retraction agents were prepared by diluting them using Dulbecco’s Modified Eagle’s Medium (DMEM, Sigma-Aldrich, St. Louis, MO, USA) to obtain 1 mg/mL and 0.5 mg/mL concentrations.

### 2.2. Cell Culture

Primary human gingival fibroblasts (HGFs) were isolated from 1–2 mm fragments of healthy gingival tissue, following the procedure patented by Dominiak and Saczko [11]. The biopsies were provided by the Department of Dental Surgery of the Faculty of Dentistry of Wroclaw Medical University, according to the requirements of the Wroclaw Medical University Bioethical Committee (approval No KB-8/2010) and the Helsinki Declaration, as revised in 2013. Gingival tissue was obtained from patients who agreed to a voluntary tissue biopsy before the planed tooth extraction. The explants were placed in a cell culture medium (DMEM, Sigma-Aldrich, St. Louis, MO, USA) and supplemented with 10% fetal bovine serum (FBS, Sigma-Aldrich, St. Louis, MO, USA) and penicillin/streptomycin (Sigma-Aldrich, St. Louis, MO, USA) immediately after being taken by a scalpel. The cells were grown on Petri dishes (60 mm) (Nunc, Roskilde, Denmark) or in 25 cm^2^ flasks (Nunc, Roskilde, Denmark) in a humidified atmosphere at 37 °C and 5% CO_2_. For experimental reasons, they were detached by trypsinization (0.25% Trypsin-EDTA, Sigma-Aldrich, St. Louis, MO, USA).

### 2.3. Cell Viability—MTT Assay

HGFs were seeded into 96-well cell culture plates (Nunc). After 24 h, the culture medium was replaced with a fresh medium containing the proper amount of the studied retraction agent (the final concentrations of 1 mg/mL and 0.5 mg/mL were obtained). The cells were incubated with the astringents for 5, 10, or 30 min. Additionally, to assess the long-term effect, a 24 h incubation was performed at a concentration of 1 mg/mL. Once it ended, the culture medium with retraction agent was replaced with a fresh DMEM and a 3-(4,5-dimethyl-2-thiazolyl)-2,5-diphenyl-2H-tetrazolium bromide (MTT) assay was used. Cells were incubated for 90 min with 100 μL of the MTT reagent (Sigma-Aldrich) at 37 °C. Then, formazan crystals were dissolved by adding 100 μL of acidic isopropanol and by mixing. The absorbance was measured at 560 nm using a multiwell plate reader (GloMax Discover Microplate Reader, Promega, Madison, WI, USA). The results are expressed as the percentage of treated cells with altered mitochondrial function in relation to untreated control cells with normal mitochondrial activity, which was considered as 100% cell viability.

### 2.4. Cell Proliferation—BrdU Assay

HGFs were seeded into 96-well cell culture plates (Nunc, Roskilde, Denmark). After 24 h, the culture medium was replaced with a fresh medium containing the proper amount of the studied retraction agent (the final concentration of 1 mg/mL was selected). The cells were incubated with the astringents for 5 min, 10 min, or 24 h. Measurement of bromodeoxyuridine (BrdU) incorporation into newly synthesised DNA strands was performed to detect actively proliferating cells. The BrdU Cell Proliferation Assay Kit (Millipore, Burlington, Massachusetts, USA) was used per the manufacturer’s instructions. The absorbance was measured at 450 nm using a multiwell plate reader (GloMax Discover Microplate Reader, Promega, Madison, WI, USA). The results are expressed as the percentage of treated cells with an altered BrdU incorporation ability compared to untreated control cells with normal ability, which was considered to be 100% cellular proliferation.

### 2.5. Cytoskeletal Organization—Zyxin and F-actin Localization by Confocal Laser Scanning Microscopy

The following immunofluorescence procedure was performed to evaluate the zyxin and F-actin distribution. Primary HGFs (1000 cells) were grown on coverslips for 24 h at 37 °C and then incubated with retraction agents diluted in the culture medium (1 mg/mL) for 24 h at 37 °C. Next, the fibroblasts were washed with PBS (BioShop, Mainway, Burlington, Canada) for 5 min at room temperature, fixed using 4% PFA (Sigma-Aldrich) in PBS (10 min at room temperature), permeabilized with 1% triton X-100 (Sigma-Aldrich, St. Louis, MO, USA) in PBS (*v*/*v*) (3 × 3 min at room temperature) and blocked with 1% FBS in PBS (1 h at 37 °C). All washing steps were performed with PBS. After overnight incubation with primary mouse monoclonal antizyxin antibody (diluted 1:200) (Abcam, Cambridge, UK) at 4 °C, the cells were washed with PBS (2 × 10 min at room temperature) and labelled with the secondary antibody—Fluorescein (FITC)-conjugated AffiniPure Fragment Donkey Anti-Mouse IgG (for 60 min at room temperature; diluted 1:100) (Jackson ImmunoResearch, Cambridgeshire, UK) mixed with Alexa 546-conjugated phalloidin (at a concentration of 2 μg/mL) (Life Technologies, Carlsbad, CA, USA). Then, the cells were mounted in a fluorescence mounting medium (DAKO). An Olympus FluoView FV1000 confocal laser scanning microscope (Olympus, Tokio, Japan) with 60x magnification was used for imaging.

### 2.6. Oxidative Stress—Nitrite (NO^2−^) Concentration Assay

The Griess Reagent System (Promega, Madison, WI, USA) was used according to the manufacturer’s instructions to measure nitrite (NO^2−^), one of two primary stable and nonvolatile breakdown products of nitric oxide (NO). The procedure was performed 24 h after the end of various time points of incubation (10 min and 24 h) of HGFs with the gingival retraction agents, diluted in the cell culture medium to 1 mg/mL concentration. The level of nitrite in the cell culture medium was analyzed spectrophotometrically. The absorbance was measured at 560 nm using a multiwell plate reader (GloMax Discover Microplate Reader, Promega, Madison, WI, USA).

### 2.7. RNA Isolation and Relative Real-Time RT-PCR

Cells were grown on Petri dishes (2 × 10^6^ of cells) for 24 h at 37 °C, and then incubated with retraction agents diluted in the culture medium (1 mg/mL) for 24 h at 37 °C. Next, the fibroblasts were gently washed with PBS (BioShop), scraped with a rubber policeman and centrifuged (280× *g*, 5 min). The dry cell pellet was stored at −20 °C for further experiments. The total RNA was isolated using a NucleoSpin RNA II kit (Macherey-Nagel & Co., Düren, Germany) following the manufacturer’s protocol. Reverse transcription reaction (RT) was performed using 600 ng of extracted total RNA and a High-Capacity cDNA Reverse Transcription Kit (Thermo Fisher Scientific, Waltham, MA, USA) in a final volume of 20 μL according to the manufacturer’s instructions. AceQ qPCR Probe Master Mix (Vazyme Biotech, Nanjing, Jiangsu, China) and specific TaqMan Assays: Hs00533490_m1 (for superoxide dismutase 1; SOD1), Hs00167309_m1 (for superoxide dismutase 2; SOD2), Hs00162090_m1 (for superoxide dismutase 3; SOD3), Hs01110250_m1 (for heme oxygenase 1; HMOX1), Hs00829989_gH (for glutathione peroxidase 1; GPX1) and Hs99999905_m1 (for glyceraldehyde-3-phosphate dehydrogenase; GAPDH) (Thermo Fisher Scientific Waltham, MA, USA) were used to assess RNA expression according to the manufacturers’ instructions. We added 3 μL of three-times-diluted RT products to a single real-time polymerase chain reaction (RT-PCR). All the reactions were performed in triplicate in 96-well plates under the following thermal cycling conditions: 5 min at 95 °C followed by 40 cycles of 10 s at 95 °C and 30 s at 60 °C. The reactions were run in the Optical Real-Time PCR Thermocycler (Biometra GmbH, Göttingen, Germany) and the threshold cycle data (Ct) were collected using qPCRsoft (Biometra GmbH, Göttingen, Germany). For relative quantification (RQ), the samples were normalized against the expression of GAPDH mRNA using the comparative Ct method (2^−ΔΔCt^).

### 2.8. Statistical Analysis

The data are presented as mean ± standard deviation. All experiments were performed in triplicate. The statistical significance of the differences between the mean values of different groups (nine astringents) and the untreated control group was evaluated by Student’s t-test using Statistica version 13.3 software (StatSoft, Dell, Round Rock, TX, USA). Values of *p* ≤ 0.05 were marked with an asterisk and considered statistically significant. Additionally, to assess the significance of the differences between various formulations (gel, paste, foam, and control), the results were analyzed by a one-way ANOVA using α = 0.05. F-values and *p*-values were determined, with the values of *p* ≤ 0.05 considered statistically significant. Tukey’s HSD test was performed when ANOVA indicated statistically significant results.

## 3. Results

### 3.1. Cell Viability

**Figure 1:**
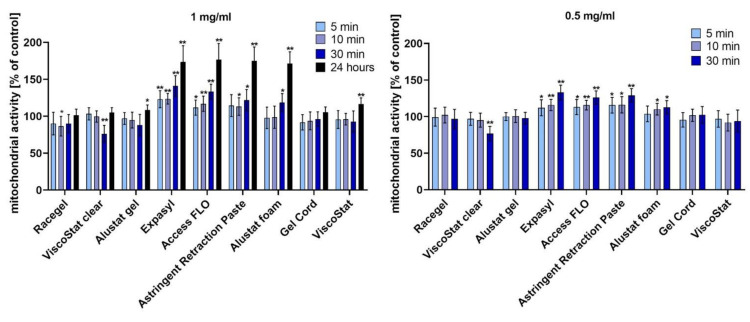
The viability evaluated by MTT assay in human gingival fibroblasts after various time points of incubation (5, 10, and 30 min, and 24 h), with the gingival retraction agents diluted in the cell culture medium to 1 mg/mL and 0.5 mg/mL concentrations; * *p* < 0.05, ** *p* < 0.005.

The results of the MTT assay are presented in Figure 1. Cell-viability evaluation based on the mitochondrial activity measurement revealed that most of the studied retraction agents were not cytotoxic to HGFs in both studied concentrations (1 and 0.5 mg/mL) and after all studied periods of incubation (5, 10, and 30 min, and 24 h). Only in the case of a 30 min incubation with ViscoStat Clear was cell viability significantly reduced, which was demonstrated by a decreased level of mitochondrial activity. Contrary to this, some of the tested astringents even increased mitochondrial activity of HGFs compared to the untreated control. Such enhancement was particularly significant in the case of three pastes containing 15% aluminium chloride and one foam containing 20% aluminium chloride.

### 3.2. Cell Proliferation

**Figure 2:**
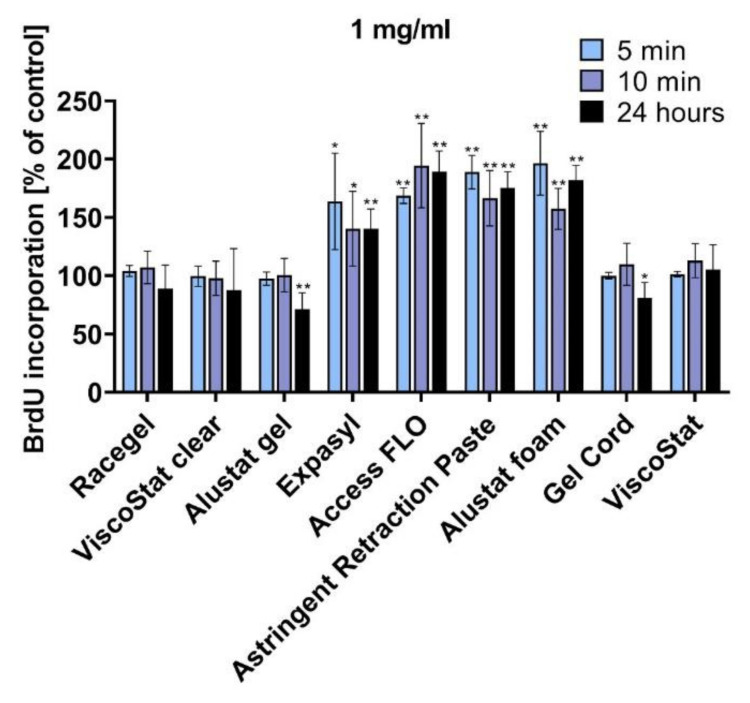
The cell proliferation detected by BrdU incorporation assay in human gingival fibroblasts after various time points of incubation (5 min, 10 min, and 24 h), with the gingival retraction agents diluted in the cell culture medium to 1 mg/mL concentration; * *p* < 0.05, ** *p* < 0.005.

Figure 2 shows the results of the BrdU incorporation assay used to evaluate cell proliferation. Similarly to the MTT assay results, the BrdU assay demonstrated the biocompatibility of most of the studied agents used in a concentration of 1 mg/mL for 5 min, 10 min, and 24 h. Only a prolonged 24 h incubation with Alustat gel and Gel cord resulted in a significant decrease of HGF proliferation (compared to the untreated control, proliferation was reduced to 68% for Alustat gel and 80% for Gel cord). This investigation confirmed the stimulating properties of pastes and foam containing aluminium chloride (Expasyl, Access FLO, Astringent Retraction Paste and Alustat foam), as cell proliferation was significantly increased after incubation with these agents when compared to the untreated control.

### 3.3. Cytoskeletal Organization—Zyxin and F-actin Localisation by Confocal Laser Scanning Microscopy (CLSM)

**Figure 3:**
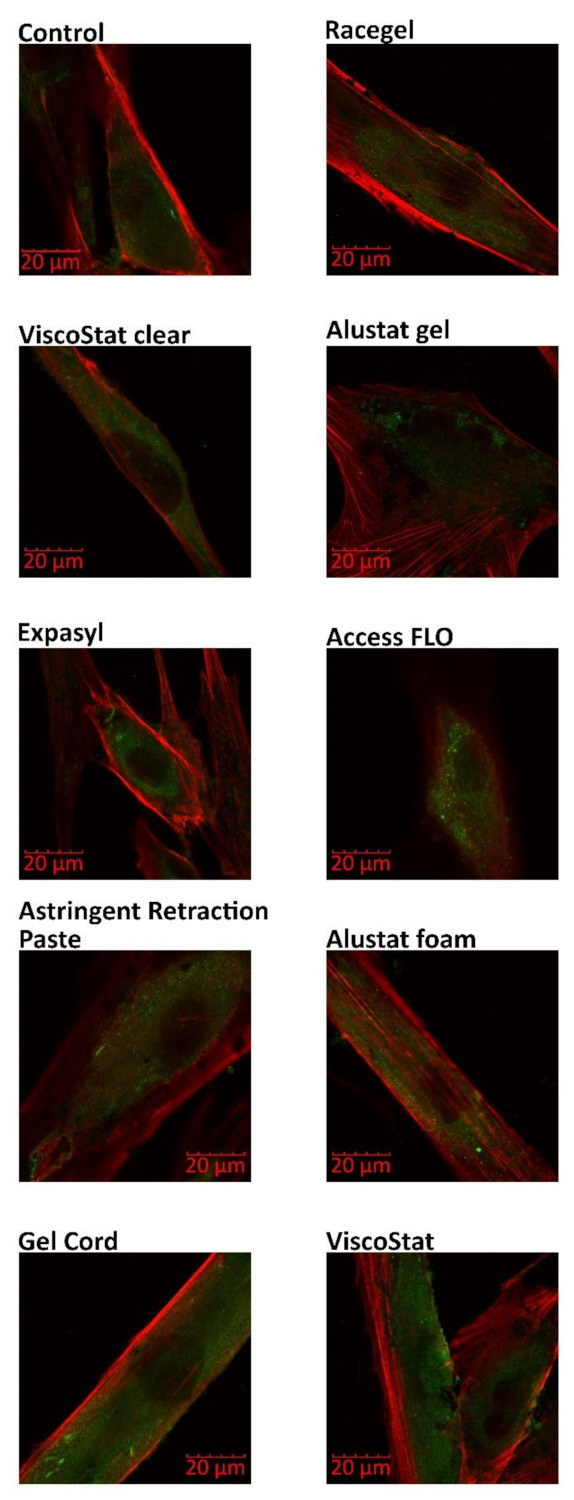
Confocal microscopy evaluation of zyxin and F-actin distribution in human gingival fibroblasts after 24 h of incubation with the gingival retraction agents diluted in the cell culture medium to 1 mg/mL concentration; green colour—zyxin, red colour—F-actin; magnification: 60×.

The distribution of two proteins responsible for cytoskeleton organization—zyxin and F-actin—was detected using immunofluorescence and visualized with the CLSM method (Figure 3). It was revealed that a prolonged 24 h incubation of HGFs with the selected astringents did not significantly influence cytoskeletal organization, since no pronounced changes of zyxin and F-actin distribution were observed upon comparing the cells incubated with the retraction agents to the untreated control cells.

### 3.4. Oxidative Stress—Nitrite (NO^2−^) Concentration Assay

**Figure 4:**
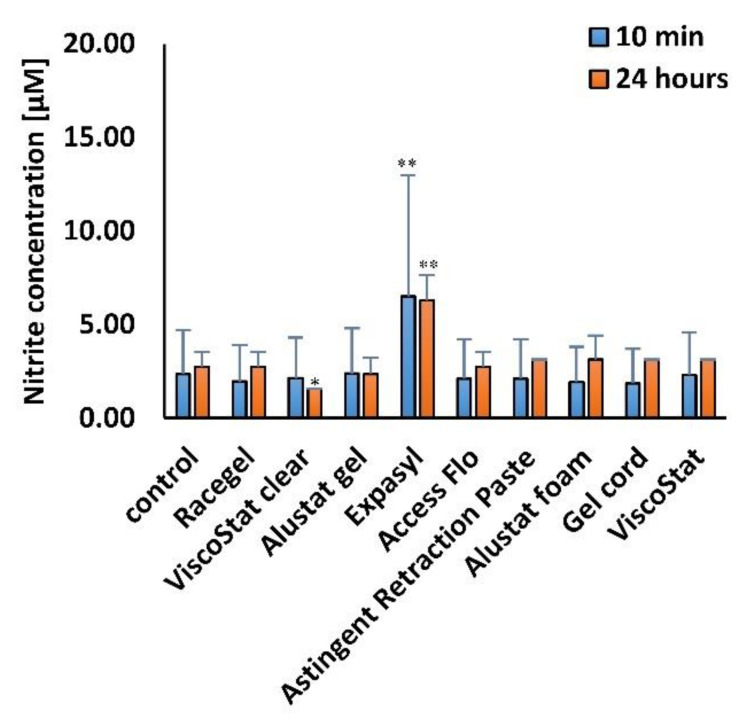
The oxidative stress detected by nitrite (NO^2−^) concentration assay in human gingival fibroblasts after various time points of incubation (10 min and 24 h), with the gingival retraction agents diluted in the cell culture medium to 1 mg/mL concentration; * *p* < 0.05, ** *p* < 0.005.

The results of nitrite (NO^2−^) concentration assay shown that only Expasyl induced oxidative stress in HGFs after incubation for both 10 min and 24 h (Figure 4). None of the other astringents tested significantly increased the level of nitrites (NO^2−^) when compared to the level detected in the untreated control cells.

### 3.5. Antioxidant Defense Systems—RNA Expression of SOD1, SOD2, SOD3, HMOX1, and GPX1

**Figure 5:**
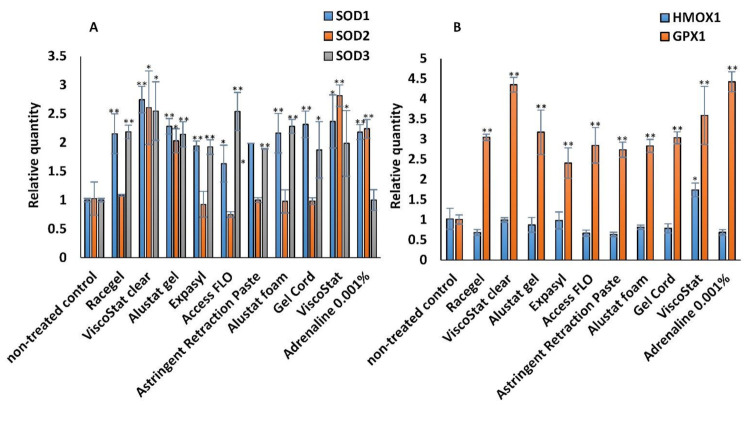
The RNA expression levels of: (**A**) SOD1, SOD2, and SOD3; and (**B**) HMOX1 and GPX1 detected by real-time RT-PCR in human gingival fibroblasts after 24 h of incubation with the gingival retraction agents diluted in the cell culture medium to 1 mg/mL concentration; * *p* < 0.05, ** *p* < 0.005.

Results of relative real-time RT-PCR analysis are presented in Figure 5 (A—for SOD1, SOD2, and SOD3; and B—for HMOX1 and GPX1). It was demonstrated that a 24 h incubation of HGFs with the selected chemical retraction agents significantly increased the relative quantity of RNA of antioxidant defense system components (SOD1, SOD3, GPX1). On the other hand, no significant influence on the RNA expression of the other antioxidant enzymes (SOD2 and HMOX1) was revealed. All results were related to those for the untreated control, for which the expression was considered to be 1. Additionally, positive control was prepared—after incubation with adrenaline 0.001%—to be compared with the results for the studied astringents. SOD3 expression was similar for both untreated and positive control, and significantly higher for the studied retraction agents. For SOD1, SOD2, HMOX1, and GPX1, the relative quantities were similar for the cells incubated with the studied retraction agents and for the positive control incubated with adrenaline (Figure 5).

### 3.6. Statistical Analysis—The Significance of Differences between Various Types of Formulations

The comparison of results for gels, pastes, foam, and the untreated control revealed that significant differences occurred, particularly between pastes, pastes and foam, and gels with astringents (Table S1). Such a tendency was observed for the results of the MTT assay and BrdU assay, as well as the RT-PCR results for SOD1, SOD3, and GPX1. The results of nitrite (NO^2−^) concentration assay, as well as RT-PCR for SOD2 and HMOX1, did not demonstrate significant dependence on the type of formulation tested.

## 4. Discussion

Astringents in the form of solutions are the most popular conventional chemical retraction/displacement agents. In dental practices, three chemical groups of gingival retraction/displacement astringents are mainly used, i.e., aluminium chloride (AC), aluminium sulphate (AS), and ferric sulphate (FS). Their clinical advantages, disadvantages, and after-effects have been reported, and their in vitro cytotoxicity has been evaluated, taking into account different parameters of gingival inflammation [5,8,9,10].

Several in vitro studies, using both established cell lines and primary cell cultures, reported a higher cytotoxicity of astringents than in vasoconstrictors [12,13,14,15,16,17,18,19]. However, different results were presented, and no consensus exists on which type of astringents has the strongest effect on the human periodontium. Kopač et al. showed that astringents in commercial solution forms were cytotoxic to Chinese hamster lung fibroblasts and primary rat keratinocytes cell cultures. Moreover, the effect of 25% AC was significantly higher than that of the other retraction chemicals [12,13]. A strong cytotoxic effect of 25% and 24.8% AC in HGFs was demonstrated by Yalcin et al. [14], while Lodetti et al. suggested strong cytotoxicity of 15.5% FS on gingival keratinocytes [15]. Liu et al. reported that 15.5% FS was the most cytotoxic, and inhibited proliferation of HGFs more than 20% AS [16]. Farzin et al. tested three astringents in gel form on a model of the normal human gingival cell line (HGF1-PI1) and concluded that 25% AC was the most toxic agent in all periods, whereas after 1 min, 20% FS showed a lower cytotoxic effect compared to 25% AS; after 5 min, both AS and FS were similarly cytotoxic; and after 15 min, AS showed lower cytotoxicity compared to FS [17]. In our previous study, we compared dynamic oxidoreduction potential of commonly used astringent retraction/displacement agents on primary HGFs, and concluded that FS-based agents were the most cytotoxic, followed by AC and AS. The cytotoxicity of these chemicals increased with the concentration, and the pharmacological form of these agents influenced cell viability. The chemicals in the form of solutions were more cytotoxic than in the form of gels [18]. Labban et al. reported that evaluated hemostatic agents (based on AC and FS) showed cytotoxicity with increasing time intervals. Astringedent (solution of 15.5% FS) showed the highest cytotoxic effect on HGFs, whereas Expasyl paste (kaolin with 15% AC) showed the least toxic effect in comparison to other chemical agents evaluated [19].

There are also several clinical studies concerning the evaluation of effectiveness and biocompatibility of different astringents used for gingival retraction. AC in 5.25–25% concentration was considered a very effective medicament. Moreover, fewer inflammatory cytokines were released in the case of cordless methods compared to packing methods [20]. However, another study revealed that in concentrations exceeding 10%, AC can cause local tissue destruction [10]. It was demonstrated that AS can inhibit intercapillary plasma-protein migration and disrupt bleeding through vasoconstriction and precipitation of tissue proteins on the superficial layer of the mucosa [7]. While such FS-based agents as coagulants minimize bleeding from periodontal microvasculature, they also can cause inflammation of gingival tissues in standard concentrations. Additionally, yellowish-brown or black discolouration of gingival and teeth tissues, as well as ceramic dental restorations, was observed [8]. Recently, Igic et al. showed that the observed clinical changes (higher gingival bleeding index (GBI) and salivary concentration of monocyte chemoattractant protein 1 (MCP-1)) were more pronounced after a chemical–mechanical gingival procedure with FS than with AC [21].

Incorporation of these chemicals into injectable retraction or retraction/impressions systems has been proposed to minimize their harmful influence on gingival tissues. Several studies evaluated the clinical effect of the new, injectable forms of retraction media with incorporated astringents on periodontal-tissue health and considered them better than that of cord techniques; however, the induced biochemical mechanisms affecting periodontal tissues status have not been completely explained [1,8,22,23]. On the other hand, some pieces of evidence suggest insufficient effectiveness of the new systems in clinical use [24]. Therefore, a more detailed study concerning the cellular effects induced upon the application of the new gingival retraction systems is necessary.

In this in vitro study, we evaluated the biological impact of three different injectable forms of gingival/retraction media (gel, paste, and foam) with three chemical groups of astringents (AC, AS, FS) on HGFs. The human oral and gingival fibroblasts are involved in the inflammatory response in acute wounds and control certain functions in the intraoral wound-healing processes. Moreover, they respond to different external stimuli, including mechanical stress [25]. Their presence in the oral mucosa lamina propria, as well as their multiple biological functions, made HGFs a common model for the evaluation of cytotoxicity of dental materials [26,27].

During retraction/displacement procedures, the astringents from injectable forms of retraction/displacement media come into contact with gingival tissues and can have a destructive effect on the gingival epithelium and connective tissues. Recently, different injectable retraction/displacement media were used, mostly gels, pastes, and foams, with various concentrations of AC, AS, and FC. In our previous study, we reported that retraction gels with astringents had a lower cytotoxic effect on HGFs compared to solutions [18]. Labban et al. showed that Expasyl paste was less cytotoxic to HGFs than solutions with astringents [19].

In the current research, we have revealed that most of the studied injectable retraction agents were not cytotoxic to HGFs. Some of the tested astringents—pastes containing 15% aluminium chloride (Expasyl, Access FLO, Astringent Retraction Paste) and Alustat foam containing 20% aluminium chloride—actually increased mitochondrial activity of HGFs compared to the untreated control. In addition, the BrdU incorporation assay confirmed the biocompatibility of the studied astringents and even the stimulating properties of pastes with 15% AC and the foam with 20% AC, which were manifested through significantly increased cell proliferation after incubation with these agents when compared to the untreated control. The superior biocompatibility of Alustat foam compared to Alustat gel was also confirmed in a study by Szymonowicz et al., in which the cytotoxicity of different astringents was compared [28].

Additionally, in the current study, no HGF cytoskeleton disruption was observed when the distribution of two proteins involved in its organization (zyxin and F-actin) was analyzed. The evaluation of zyxin distribution is particularly interesting due to its involvement in the response to mechanical tension and its role in the facilitation of actin polymerization [29]. To the best of our knowledge, this is the first study focused on this topic. In our previous studies, we investigated the cytotoxic effects of vasoconstrictive retraction/displacement agents. In contrast to the currently assessed astringents, some vasoconstrictors significantly influenced cell structure, leading to disruption of actin filaments and microtubules [30], as well as disturbance of fibronectin expression [31].

Our previous study also revealed that the levels of oxidative changes (lipid peroxidation and protein damage) and SOD2 expression after application of vasoconstrictive agents in gel formulations were lower than after using commercial agents in the form of solutions [30]. In the current research, we detected a similar level of nitrite—one of the markers of oxidative stress—in cells incubated with different astringents and in the untreated control. Only the exposure to Expasyl significantly elevated the level of nitrites. One possible explanation could be related to the finding reported by Dederichs et al., who revealed that compared to all other retraction materials studied, Expasyl generated the highest pressure on the novel gingival sulcus model, which could result from its consistency [32].

We assessed the RNA expression of selected enzymes to enable a more detailed elucidation of antioxidant mechanisms activated in HGFs upon incubation with astringents. The chemical retraction agents evaluated significantly increased the relative quantity of RNA of SOD1, SOD3, and GPX1. On the other hand, no significant influence on the RNA expression of the other antioxidant enzymes (SOD2 and HMOX1) was revealed. All enzymes studied are significant components of the antioxidant defense system, since superoxide dismutases (SODs) detoxify the superoxide radicals into hydrogen peroxide and oxygen [33], glutathione peroxidase 1 (GPX1) catalyses the reduction of H2O2 or organic hydroperoxides to water or the corresponding alcohols [34], and heme oxygenase 1 (HMOX1) catalyses the first step of the oxidative degradation of the heme group and is involved in the suppression of the inflammatory response [35]. It could be concluded that selected antioxidant responses were induced to eliminate reactive oxygen species and protect cells against destruction caused by oxidative stress. Interestingly, the RNA expression of SOD2 and HMOX1, both of which could be located in mitochondria, was not influenced by astringents. These results support the rationale of the evaluation of several different oxidative stress markers. Furthermore, considering all the results obtained, it could be concluded that HGFs were stimulated by incubation with astringents to activate defense systems protecting them against oxidative damage, and that these systems were capable of resisting harmful effects.

## 5. Conclusions

The results obtained in this study suggest good biocompatibility of the new formulations of chemical retraction agents with primary HGFs from human gingiva. The characterization of biological response induced in HGFs by injectable forms of retraction astringents confirmed that such materials are safe for human periodontal tissues. In a further step, their clinical effects should be assessed to select the safest and most effective agents for the free gingival margin retraction/displacement procedure. It could be expected that through a better understanding of the properties of retraction agents, better clinical outcomes of the gingival margin retraction procedure could be achieved.

## Figures and Tables

**Table 1:** Characteristics of the studied gingival retraction/displacement agents.

Trade Name	Manufacturer	Part or Batch Number	Active Ingredient	Clinical Form
**Racegel**	Septodont (Cedex, France)	B16792AC	25% aluminium chloride	gel
**ViscoStat clear**	Ultradent Products, Inc. (South Jordan, UT, USA)	BB68F	25% aluminium chloride	gel
**Alustat gel**	Cerkamed (Nisko, Poland)	2711171	25% aluminium chloride	gel
**Expasyl**	KerrHawe SA (Bioggio, Switzerland)	7503	15% aluminium chloride	paste
**Access FLO**	Centrix (Shelton, CT, USA)	A42959	15% aluminium chloride	paste
**Astringent Retraction Paste**	3M ESPE (St. Paul, MN, USA)	3472601	15% aluminium chloride	paste
**Alustat foam**	Cerkamed (Nisko, Poland)	2205171	20% aluminium chloride	foam
**Gel Cord**	Pascal International (Bellevue Washington, USA)	100119E	25% aluminium sulphate	gel
**ViscoStat**	Ultradent Products, Inc. (South Jordan, UT, USA)	BB68F	20% ferric sulphate	gel

## Data Availability

All data presented in this study are included in the published article or are available on request from the corresponding author.

## Supplementary Materials

The following are available online at https://www.mdpi.com/article/10.3390/ma14082081/s1, Table S1: Results of a post hoc Tukey’s HSD test showing the significance of differences between different types of formulation (gel, paste, and foam) and the untreated control, revealed in various assays; red colour denotes statistically significant differences (*p*-value < 0.05).
