# Supplementary material for: Biological Response Induced in Primary Human Gingival Fibroblasts upon Exposure to Various Types of Injectable Astringent Retraction Agents
Source: Materials (Basel). 2021 Apr 20;14(8):2081. doi: 10.3390/ma14082081 (PMC8074361; doi:10.3390/ma14082081)
Supplement: Supplementary file 1 [file materials-14-02081-s001.zip › materials-1182802-supplementary.pdf]

materials

Article

Biological Response Induced in Primary Human Gingival Fibroblasts upon Exposure to Various Types of Injectable Astringent Retraction Agents

Danuta Nowakowska [1], Julita Kulbacka [2], Joanna Wezgowiec [3,*], Anna Szewczyk [2], Dagmara Baczynska [2], Marek Zietek [3], Wlodzimierz Wieckiewicz [1] and Jolanta Saczko [2]

[1] Department of Prosthetic Dentistry, Wroclaw Medical University, Wroclaw 50-425, Poland; danuta.nowakowska@umed.wroc.pl (D.N.); wlodzimierz.wieckiewicz@umed.wroc.pl (W.W.)
[2] Department of Molecular and Cellular Biology, Wroclaw Medical University, Wroclaw 50-556, Poland; julita.kulbacka@umed.wroc.pl (J.K.); a.szewczyk@umed.wroc.pl (A.S.); dagmara.baczynska@umed.wroc.pl (D.B.); jolanta.saczko@umed.wroc.pl (J.S.)
[3] Department of Experimental Dentistry, Wroclaw Medical University, Wroclaw 50-425, Poland; marek.zietek@umed.wroc.pl
* Correspondence: joanna.wezgowiec@umed.wroc.pl

Supplementary Table S1. Results of a post-hoc Tukey's HSD tests showing significance of differences between different types of formulation (gel, paste and foam) and the untreated control, revealed in various assays; red color denotes statistically significant differences (p-values < 0.05).

MTT 1:10 5 min

F(3, 85)=17.770, p=0.00000

Tukey's HSD test; MS = 146.74, df = 85.000

	control	gel	paste	foam
control		0.861889	0.036013	0.987731
gel	0.861889		0.000145	0.948708
paste	0.036013	0.000145		0.000615
foam	0.987731	0.948708	0.000615	

MTT 1:10 10 min

F(3, 84)=26.677, p=0.00000

Tukey's HSD test; MS = 122.50, df = 4.000

	control	gel	paste	foam
control		0.696372	0.007736	0.996533
gel	0.696372		0.000146	0.670467
paste	0.007736	0.000146		0.000201
foam	0.996533	0.670467	0.000201	

MTT 1:10 30 min

F(3, 76)=53.978, p=0.0000

Tukey's HSD test; MS = 183.16, df = 6.000

	control	gel	paste	foam
control		0.289465	0.000190	0.087235
gel	0.289465		0.000148	0.000149
paste	0.000190	0.000148		0.080633
foam	0.087235	0.000149	0.080633	

MTT 1:20 5 min

F(3, 85)=15.726, p=0.00000

Tukey's HSD test; MS = 96.388, df = 85.000

	control	gel	paste	foam
control		0.961144	0.026021	0.899823
gel	0.961144		0.000145	0.306897
paste	0.026021	0.000145		0.035172
foam	0.899823	0.306897	0.035172	

MTT 1:20 10 min

F(3, 86)=22.907, p=0.00000

Tukey's HSD test; MS = 85.732, df = 86.000

	control	gel	paste	foam
control		0.982420	0.003398	0.190645
gel	0.982420		0.000145	0.002470
paste	0.003398	0.000145		0.331595
foam	0.190645	0.002470	0.331595	

MTT 1:20 30 min

F(3, 84)=51.392, p=0.0000

Tukey's HSD test; MS = 147.90, df = 84.000

	control	gel	paste	foam
control		0.678830	0.000162	0.245380
gel	0.678830		0.000146	0.000372
paste	0.000162	0.000146		0.003408
foam	0.245380	0.000372	0.003408	

BrdU 1:10 5 min

F(3, 32)=72.655, p=0.00000

Tukey's HSD test; MS = 271.11, df = 32.000

	control	gel	paste	foam
control		0.999954	0.000165	0.000165
gel	0.999954		0.000165	0.000165
paste	0.000165	0.000165		0.407340
foam	0.000165	0.000165	0.407340	

BrdU 1:10 10 min

F(3, 139)=69.572, p=0.0000

Tukey's HSD test; MS = 614.28, df = 139.00

	control	gel	paste	foam
control		0.977717	0.000008	0.003966
gel	0.977717		0.000008	0.000008
paste	0.000008	0.000008		0.001996
foam	0.003966	0.000008	0.001996	

BrdU 1:10 24 h

F(3, 70)=74.708, p=0.0000

Tukey's HSD test; MS = 601.92, df = 70.000

	control	gel	paste	foam
control		0.709024	0.000156	0.000152
gel	0.709024		0.000150	0.000150
paste	0.000156	0.000150		0.461918
foam	0.000152	0.000150	0.461918	

Griess 1:10 10 min

F(3, 74)=2.1411, p=0.10224

Tukey's HSD test; MS = 4.3961, df = 74.000

	control	gel	paste	foam
control		0.999092	0.483873	0.985844
gel	0.999092		0.101339	0.990776
paste	0.483873	0.101339		0.291148
foam	0.985844	0.990776	0.291148	

Griess 1:10 24 h

F(3, 36)=3.6336, p=0.02178

Tukey's HSD test; MS=1.5382, df = 36.000

	control	gel	paste	foam
control		0.995641	0.274672	0.970410
gel	0.995641		0.013002	0.852025
paste	0.274672	0.013002		0.575156
foam	0.970410	0.852025	0.575156	

MTT 1:10 24 h

F(3, 245)=456.16, p=0.0000

Test HSD

	control	gel	paste	foam
control		0.628177	0.000008	0.000008
gel	0.628177		0.000008	0.000008
paste	0.000008	0.000008		0.654788
foam	0.000008	0.000008	0.654788	

SOD1 1:10 24 h

F(3, 26)=20.704, p=0.00000

Tukey's HSD test; MS = 0.08437, df = 26.000

	control	gel	paste	foam
control		0.000169	0.001002	0.000385
gel	0.000169		0.001491	0.667385
paste	0.001002	0.001491		0.400421
foam	0.000385	0.667385	0.400421	

SOD2 1:10 24 h

F(3, 26)=6.1116, p=0.00274

Tukey's HSD test; MS = 0.38501, df = 26.000

	control	gel	paste	foam
control		0.139937	0.988357	0.999728
gel	0.139937		0.003615	0.110984
paste	0.988357	0.003615		0.996891
foam	0.999728	0.110984	0.996891	

SOD3 1:10 24 h

F(3, 26)=8.6511, p=0.00038

Tukey's HSD test; MS = 0.14010, df = 26.000

	control	gel	paste	foam
control		0.000423	0.000855	0.001672
gel	0.000423		0.997986	0.943240
paste	0.000855	0.997986		0.916860
foam	0.001672	0.943240	0.916860	

HMOX1 1:10 24 h

F(3, 26)=1.3521, p=0.27928

Tukey's HSD test; MS = 0.10598, df = 26.000

	control	gel	paste	foam
control		0.999991	0.629703	0.865304
gel	0.999991		0.269913	0.767636
paste	0.629703	0.269913		0.994048
foam	0.865304	0.767636	0.994048	

GPX1 1:10 24 h

F(3, 26)=20.451, p=0.00000

Tukey's HSD test; MS = 0.25487, df = 26.000

	control	gel	paste	foam
control		0.000169	0.000370	0.000945
gel	0.000169		0.006149	0.253515
paste	0.000370	0.006149		0.957308
foam	0.000945	0.253515	0.957308	
